# Rumination and Performance in Dynamic, Team Sport

**DOI:** 10.3389/fpsyg.2015.02016

**Published:** 2016-01-08

**Authors:** Michael M. Roy, Daniel Memmert, Anastasia Frees, Joseph Radzevick, Jean Pretz, Benjamin Noël

**Affiliations:** ^1^Department of Psychology, Elizabethtown CollegeElizabethtown, PA, USA; ^2^North-West UniversityPotchefstroom, South Africa; ^3^German Sport UniversityCologne, Germany; ^4^Gettysburg CollegeGettysburg, PA, USA

**Keywords:** sport, rumination, reflection, creativity

## Abstract

People high in rumination are good at tasks that require persistence whereas people low in rumination is good at tasks that require flexibility. Here we examine real world implications of these differences in dynamic, team sport. In two studies, we found that professional male football (soccer) players from Germany and female field hockey players on the US national team were lower in rumination than were non-athletes. Further, low levels of rumination were associated with a longer career at a higher level in football players. Results indicate that athletes in dynamic, team sport might benefit from the flexibility associated with being low in rumination.

## Introduction

The personalities of athletes and non-athletes diverge in a number of meaningful ways. For example, successful athletes tend to be higher in conscientiousness and extraversion and lower in neuroticism than are non-athletes (see Allen et al., 2013; Allen and Laborde, 2014, for review). Additionally, athletes and non-athletes differ in the ability to focus their attention (Hüttermann et al., 2013, 2014) and in their motivational outlook (Plessner et al., 2009; Memmert et al., 2015). These kinds of contrasts might extend to how susceptible athletes are to certain types of thought patterns, especially rumination.

Rumination is a thought style where people get stuck in repetitive, negative thoughts and is often linked with depression (Trapnell and Campbell, 1999; Nolen-Hoeksema et al., 2008). However, it appears that having a ruminative thought style might be beneficial in certain situations, such as when it is important to be persistent (Altamirano et al., 2010) or creative (Verhaeghen et al., 2005, 2014; Cohen and Ferrari, 2010). Given that persistence might also be needed for the mastery of athletic skills (Ericsson et al., 1993) and that creativity is important to athletes (Memmert and Roth, 2007; Memmert, 2011, 2015a,b), high levels of rumination might be related to success in dynamic, team sport. Alternatively, since it is important for athletes in team sport to be able to quickly shift focus during competition (Geisler and Leith, 1997), the goal-switching ability that is associated with low rumination (Altamirano et al., 2010) might also benefit these athletes. We examined whether athletes competing in football (soccer) and field hockey were lower or higher than non-athletes in rumination and whether or not rumination was related to success and longevity in their sport.

### Rumination

Rumination is intense self-reflection with repetitive, rigid thoughts that amplify negative personal experiences that may lead to depression (Lyubomirsky et al., 1998; Trapnell and Campbell, 1999; Nolen-Hoeksema et al., 2008; Watson et al., 2012). Even though being mired in these recursive thoughts can prove maladaptive, this need not be the case. People high in dispositional rumination performed significantly better than low ruminators on a goal-maintenance task, a modified Stroop task, but did worse on a goal-switching task, an alternating letter-naming task (Altamirano et al., 2010). Ruminators appear to be more proficient at maintaining focus on tasks that require perseverance, but less adept at tasks that require switching focus.

However, the consequences of rumination may also depend on the specific type of rumination involved. Rumination can be broken down into the subcomponents of brooding and reflective rumination (Treynor et al., 2003; Burwell and Shirk, 2007). Brooding rumination, which has been linked with depression, involves repeated negative thoughts that are more emotional in nature (Roberts et al., 1998; Treynor et al., 2003). Reflective rumination involves repeatedly thinking about a negative situation in a manner that is more contemplative and active in an attempt to assess and solve the problem (Roberts et al., 1998; Treynor et al., 2003; Burwell and Shirk, 2007; Schoofs et al., 2010).

Having a reflective ruminative thought style might be beneficial to creativity, traditionally defined as the production of ideas that are both novel and appropriate (Kaufman, 2009). People high in reflective rumination are more serious about their creative endeavors and exhibit greater overall creativity (Verhaeghen et al., 2005, 2014; Cohen and Ferrari, 2010). In particular, musicians tend to be higher in reflective rumination, and high levels of reflective rumination are related to greater musical ability (Jones et al., 2014). Furthermore, the dual pathway model to creativity indicates that both flexibility (creating many unrelated ideas) and persistence (pursuing one category of ideas in depth) can lead to creativity (Nijstad et al., 2010). Higher reflective rumination, like that found in musicians, may lead to creativity through the persistence pathway.

### Dynamic, Team Sport

Though previous research has examined the relationship between a ruminative thought style and creativity in the arts, we are unaware of any research examining the impact of rumination in other activities such as sport. Football (Study 1) and field hockey (Study 2) provide an interesting test case for the potential benefits and detriments related to being high in rumination. Based on previous findings, opposing predictions could be made as to why a reflective mindset may or may not be helpful to athletes in these sports. For instance, being proficient at a sport, like music, requires a large amount of deliberate practice (Ericsson et al., 1993). The ability to focus thought and practice on developing a specific skill through repetition is similar to the thought pattern found in rumination (Jones et al., 2014). Being high in reflective rumination and having a singular mindset might make athletes better able to focus on improving their motor and cognitive skills. Further, being high in reflective rumination might be linked with greater creativity on the field, which has been linked with success in sports such as football (Memmert and Roth, 2007; Memmert, 2011, 2015a,b). Athletes, like musicians, might benefit from being high in persistence and therefore rumination.

Alternatively, sports competitions consist of continually shifting events where athletes are forced to quickly adjust their plans and focus, indicating that they might benefit more from the ability to be flexible. This is especially true in dynamic, team sport such as football and field hockey where numerous participants on both teams influence and change the flow of the match. Therefore, being low in rumination and better able to shift from task to task (Altamirano et al., 2010) might be more beneficial to athletes in dynamic, team sport. If the athletes were stuck thinking about a previous play, then their performance on the current play could be negatively impacted.

In addition, being high rumination might lead athletes to repetitively focus on other aspects of their performance that are not helpful during competition. For example, people high in rumination might be more susceptible to the detrimental effects of anxiety and fatigue because they are not able to stop thinking about them (Lyubomirsky et al., 1998; Watson et al., 2012). Explicitly learned skills, such as athletics, suffer greatly from anxiety (e.g., Mullen et al., 2007) and fatigue (e.g., Masters et al., 2008). The negative thought patterns associated with anxiety are likely to be amplified by rumination (Lyubomirsky et al., 1998; Watson et al., 2012), further decreasing performance.

Here we examined whether athletes in dynamic, team sport experienced either high or low levels of rumination. These athletes could have benefited from either the persistence that is associated with being high in rumination or the flexibility that is associated with being low in rumination.

## Study 1

In Study 1, we examined rumination levels in football (soccer) players. Rumination levels in professional athletes, club level athletes and non-athletes were compared to look at not only differences between athletes and non-athletes but also between athletes that had achieved different levels of success.

### Method

#### Participants

Participants were 101 men (Age *M* = 26.1, *SD* = 5.0) recruited based on their football experience from a large German University’s participant database. Twenty-five participants were professional football players (Age *M* = 22.6, *SD* = 3.7), earning their living playing football (years playing professionally *M* = 3.4, *SD* = 3.0). Forty-six participants were club football players (Age *M* = 26.0, *SD* = 4.8), with acceptance onto and continued participation on competitive club teams based on performance (years playing at the club level *M* = 11.8, *SD* = 6.3). Thirty participants (Age *M* = 29.3, *SD* = 4.1) in the control group had no football experience. Unfortunately, participants in all three groups differed in age, *F*(2,98) = 15.8, *p* < 0.001, η^2^ = 0.24 (all pairwise comparisons were significant using Fisher’s LSD). However, results did not change when age was added as a covariate to our analyses.

As men and women do tend to differ in rumination levels (Nolen-Hoeksema, 2001), we decided to limit participants in all groups to men because there was only one woman in the original sample of football players (who played at the club level). The institutional ethics board approved the research and informed consent was obtained from every participant before commencing the experiment in accordance with the Helsinki Declaration of 1975.

### Procedure

Participants completed 10 items from the Ruminative Response Scale (RRS; Treynor et al., 2003), which consisted of 5-item subscales aimed at brooding rumination (Cronbach’s α = 0.73 for these participants; example question: how often do you think “what am I doing to deserve this?”) and reflective rumination (α = 0.69; example question: how often do you go away by yourself and think about why you feel the way you do). The RRS is a widely used and well-validated measure of rumination that has been used with a number of populations (see Nolen-Hoeksema et al., 2008). However, this scale does not appear to have been used previously with athletes. A German translation of the scale was used for Study 1 (Kuehner and Weber, 1999). Separate one-way analysis of variances were used to compare the levels of reflective and brooding rumination in professional athletes, club level athletes and non-athletes (control group).

Participants also reported demographic information as well as their years of experience in football at their respective level of competition. Participants completed all of these measures individually online along with additional personality measures unrelated to this study in sessions lasting 12–15 min.

We also examined whether or not level of rumination was related to the success of the football players. Level of competition achieved by the athlete was compared, with professional athletes considered more successful than club athletes. As an additional measure of success, we measured the years of experience football players had at a given level of competition (professional or club). Long-term success in sport can be assessed in terms of career duration (cf. Ericsson et al., 1993). Given that club and professional athletes tended to differ in age and years of experience, the correlations between years of experience and rumination were analyzed separately for each of these groups. As number of years of experience and age were confounded, correlational analyses that took into account age were added.

### Results and Discussion

**FIGURE 1:**
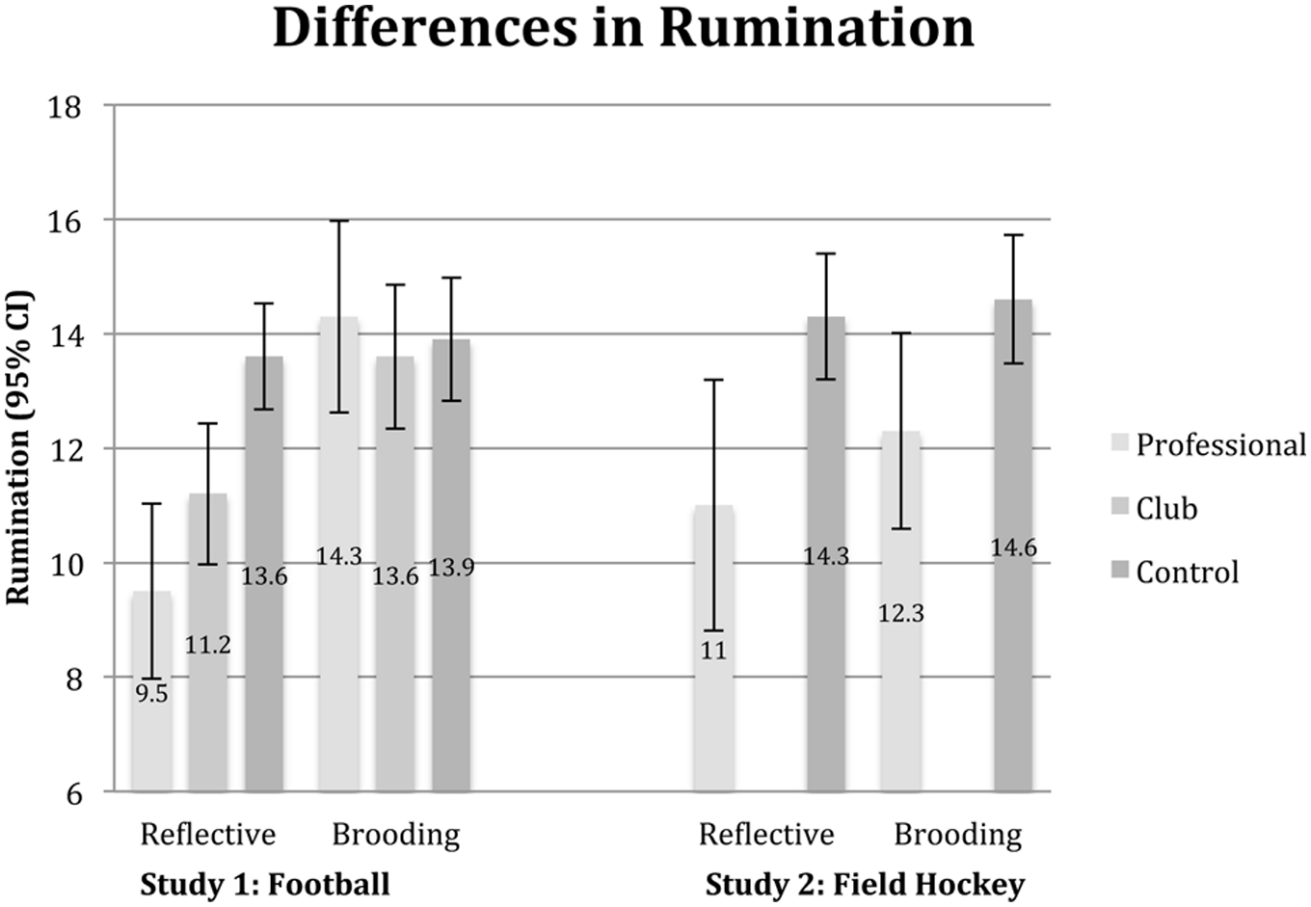
**Mean reflective and brooding rumination scores with 95% confidence intervals for athletes and control group members**.

A one-way analysis of variance indicated that reflective rumination levels differed between the control, club athlete and professional athlete groups, *F*(2,98) = 9.1, *p* < 0.001, η^2^ = 0.16. The relationship between ability level (none, club and professional) and reflective rumination produced a significant linear trend (weighted), *F*(1,98) = 17.8, *p* < 0.001, η^2^ = 0.15, with reflective rumination decreasing as ability increased (see **Figure 1**). *Post hoc* analyses using Fisher’s LSD indicated that participants in the control group (*M* = 13.6, *SD* = 2.5) had significantly higher reflective rumination levels than did club level athletes (*M* = 11.2, *SD* = 4.2; *p* = 0.005, 95% CI [0.75, 4.14]) and professional athletes (*M* = 9.5, *SD* = 3.7, *p* < 0.001, 95% CI [2.16, 6.08]). The difference between club and professional athletes was not significant, but suggested a trend in the predicted duration (*p* = 0.067, 95% CI [-0.12, 3.47] and was part of the previous significant linear trend analysis).

There was no difference between control (*M* = 13.9, *SD* = 2.9), club (*M* = 13.6, *SD* = 4.2) and professional (*M* = 14.3, *SD* = 4.1) participants in brooding rumination, *F*(2,97) = 0.31, *p* = 0.73, η^2^ = 0.01.

The relationship between rumination and number of years of experience were examined within both the professional and club level groups. The number of years competing at the professional level was marginally related to lower levels of reflective rumination, *r*(25) = -0.39, *p* = 0.053, and significantly related to lower levels of brooding rumination, *r*(25) = -0.47, *p* = 0.018. Accounting for age first (in a multiple regression analysis) did not change the results (β = -0.43 for reflection and β = -0.57 for brooding). Even though number of years competing at a professional level and age were confounded, age did not account for the results. For professional athletes, a longer career was associated with lover levels of rumination. However, for participants who competed at the club level, a longer career was not significantly related to reflective rumination, *r*(46) = 0.17, *p* = 0.36, and seemed to show a slight pattern in the opposite direction in regards to brooding rumination, *r*(45) = 0.26, *p* = 0.08.

The pattern of results found for football players was opposite to that found for musicians (Jones et al., 2014) and people high in artistic creativity (Verhaeghen et al., 2005, 2014). Football players were lower in reflective rumination than were non-athletes, and athletes lowest in reflective rumination tended to be more likely to achieve higher levels of success for longer periods of time. Overall, results indicate that being low in reflective rumination was associated with a career in football. It is possible that the ability to switch quickly from task to task that is found in people low in rumination (Altamirano et al., 2010) is linked to higher levels of success in football.

## Study 2

In Study 2 we attempted to replicate and extend the results of Study 1 with high-level athletes of a second dynamic, team sport (see recent replication recommendations by Simmons et al., 2011; Cumming, 2014; Lakens and Evers, 2014). Rumination levels in women’s field hockey players on the United States national team were compared to those of a control group. To examine if the benefits of being low in rumination might have been linked to the ability to shift attention away from previous plays, the importance the athletes placed on focusing on current play was also examined.

### Method

#### Participants

Participants were 99 women (Age *M* = 25.4, *SD* = 2.2) from the United States. Twenty-one members of the United States national women’s field hockey team (Age *M* = 25.1, *SD* = 2.1) participated. There are 30 members of the national team, but only 21 were with the team at the time of testing. The athletes received no compensation for taking part in the study.

Data from the field hockey players were compared to those of 78 women in the same age range (Age *M* = 25.6, *SD* = 2.3) recruited from the participant pool of Amazon Mechanical Turk. Mechanical Turk is a site where participants are paid for their participation in various tasks (see Buhrmester et al., 2011, for more on Mechanical Turk). The control group members were selected from a larger online sample to match the athletes in terms of gender and age. We paid control participants $1 for completing the study. Unfortunately, measure of athletic involvement was not available for this sample. However, given that Mechanical Turk participants tend to be representative of the general population (Buhrmester et al., 2011), it can be assumed that the proportion of professional athletes in the sample would be very small or non-existent.

The institutional ethics board approved the research and informed consent was obtained from every participant before commencing the experiment in accordance with the Helsinki Declaration of 1975.

#### Measures

Rumination was measured using the RRS subscales for reflective (α = 0.81) and brooding rumination (α = 0.82). As one measure of success and expertise, we utilized coaches’ ratings of the quality of the athlete’s on-field decision-making made on a scale of 1 (*low*) to 10 (*high*). The head and assistant coach rated the players performance based on their perceived likelihood of making an appropriate decision at any given time during a game. In addition, similar to the longevity measure used in the previous study, we examined the number of international matches played by the athlete (available on team website).

An athlete’s tendency to focus on past, current or future play was assessed from responses to scenarios that described offensive and defensive situations resulting in a negative outcome. Both examples described plays that happened in high-level competition (either an international game – case scenario 1- or collegiate game – case scenario 2 – see Supplementary Material for the scenario details). Participants were supplied with a transcription of the play and asked to imagine themselves as having been involved. Two questions about the plays were concerned with how much the participants would continue to think about the play after it was over (how much they would continue to analyze that play and specifically their role in the play) and two questions about how much they would think about how the game would unfold (how the team and individual would perform in the rest of the match). All questions were on a five-point scale ranging from 1 (*almost never thinking about it*) to 5 (*almost always thinking about it*). Single-sample *t*-tests were used to determine whether participants tended to fall above (almost always) or below (almost never) the midpoint of the scale.

#### Procedure

Field hockey players completed paper and pencil questionnaires for the rumination and focus items while control participants completed the RSS using an online survey platform. There does not appear to be much difference between data collected in person and data collected online through Mechanical Turk (Buhrmester et al., 2011). Participants in both groups also completed personality questionnaires for other studies. It took participants approximately 10–20 min to complete the study. Rumination levels for the professional athletes and control group members were compared using independent groups *t*-tests.

The head and assistant coach for the field hockey team rated each athlete on the quality of her decision-making (except one participant that was new to the team) with a high level of agreement in their ratings (*ICC* = 0.79). The measures of expertise (coach ratings) and experience (number of international matches) were correlated with rumination levels for the athletes.

### Results and Discussion

Field hockey players exhibited lower levels of reflective rumination (*M* = 11.0, *SD* = 4.8) than did the control group members (*M* = 14.3, *SD* = 3.1), *t*(97) = 2.74, *p* = 0.007, *d* = 0.67, 95% CI [0.89, 5.60], as found in Study 1. Unlike Study 1, there was also a difference between field hockey players (*M* = 12.3, *SD* = 3.7) and control group members (*M* = 14.6, *SD* = 4.9) in brooding rumination, *t*(97) = 1.99, *p* = 0.048, *d* = 0.53, 95% CI [0.02, 4.59], (see **Figure 1**).

Responses to the scenarios indicated the amount of anticipated focus on either the past or the future after a negative play. For analysis, the two questions about past focus and the two questions about future focus for each of the two scenarios were averaged together. On the five-point scale, participants indicated that they would not continue to think about and analyze the previous play after it was over (*M* = 2.0, *SD* = 0.83). The average score fell significantly below the midpoint of the scale (3: placing them on the side of the scale anchored by *“almost never thinking about it”*), *t*(20) = -5.29, *p* < 0.001, *d* = -1.15, 95% CI [-1.33, -0.58]. Similarly, participants indicated that they were unlikely to think about how the game would unfold (*M* = 1.4, *SD* = 0.38), also falling significantly below the midpoint of the scale, *t*(20) = -19.1, *p* < 0.001, *d* = -4.18, 95% CI [-1.77, -1.42]. Athletes believed that they would focus on the current play and not think about either the past or the future following a negative play. However, they would be more likely to think about past play than future play, *t*(20) = 4.24, *p* < 0.001, *d* = 0.92, 95% CI [0.32, 0.95].

Although the previous study indicated that rumination and expertise were related, the results for the relationship between the measure of expertise used here (coaches’ ratings of on field decision making) and rumination were not significant: reflective rumination; *r*(20) = -0.12, *p* = 0.63, and brooding rumination; *r*(20) = -0.33, *p* = 0.16. Similarly, there was no significant relationship between number of international matches played and either reflective rumination, *r*(20) = -0.01, *p* = 0.98, or brooding rumination, *r*(20) = -0.26, *p* = 0.26. Coaches’ ratings were related to the number of international games played, *r*(20) = 0.61, *p* = 0.005, indicating that amount of experience was an acceptable measure of ability. Even though there was no relationship between ratings of expertise and rumination, it should be noted that the correlational analysis reported here was not necessarily a strong test of the relationship because the sample of field hockey players contained limited variability – all had made the national team.

Field hockey players tended to be lower than non-athletes in their levels of reflective and brooding rumination. Results indicate that low rumination levels might be due to the demands of the sport; Participants indicated that they would not be likely to entertain thoughts about either the past or the future during competition.

## General Discussion

Results suggest that participation in dynamic, team sport is related to being low in reflective rumination. Professional football and field hockey players reported much lower levels of reflective rumination than did non-athletes (approximately a one standard deviation difference on average), with a similar pattern of results found in samples that differed not only in sport, but also country and gender. Moreover, football players that competed at the highest level for the longest period of time tended to have the lowest levels of reflective rumination.

Additionally, field hockey players were also lower than non-athletes in brooding rumination (approximately half a standard deviation difference). Further, professional football players that were lower in brooding rumination tended to have longer careers. It is possible that being low in both types of rumination are related to success in the career of athletes in a dynamic, team sport. It may be that avoiding repeatedly re-experiencing negative emotions associated with a previous event is beneficial to athletes. It is also possible that the lower levels of brooding rumination found in the field hockey players is related to gender differences in the two samples as women and men do tend to differ in rumination levels (Nolen-Hoeksema, 2001).

The results for athletes provide an interesting contrast to those found for musicians. Undergraduate music majors were higher than other students in reflective rumination and higher levels of reflective rumination were tied to higher ratings of performance by a panel of expert judges (Jones et al., 2014). This pattern was replicated in a large, diverse online sample with increased levels of reflective rumination related to higher self-ratings of musical expertise (Roy et al., in press). The opposite pattern seems to apply to athletes in dynamic, team sport, even though success for both athletes and musicians is tied to extensive, repetitive practice (Ericsson et al., 1993; Macnamara et al., 2014). It is specifically the more cognitive type of repetitive thought pattern found in reflective rumination (Treynor et al., 2003) that differentiates athletes and musicians (and creative people in general; Verhaeghen et al., 2005, 2014). Results indicate that the type of creativity that might make someone successful in dynamic, team sport (Memmert and Roth, 2007; Memmert, 2011, 2015a,b) is likely to be qualitatively different than the type of creativity needed in artistic pursuits (see Silvia et al., 2009, for a discussion on different types of creativity). These findings are also in line with the dual pathway model of creativity, which holds that creativity can result from either flexibility or persistence (Nijstad et al., 2010). Whereas persistence appears to be more important for artistic creativity, flexibility appears to be more important for athletic creativity.

Even though athletes and musicians are similar in their need to use repetitive practice to perfect certain skills, how those skills are put to use can be very different. In dynamic sport such as football and field hockey, conditions and situations change rapidly. It is necessary for football players to quickly forget the last play and move on to the next play (Geisler and Leith, 1997). In support of this notion, field hockey players in Study 2 reported that they would be unlikely to think about past or future plays during a match. The low reflective rumination observed in successful field hockey and football players therefore could reflect that people who are low in rumination do best on tasks that require quick shifts in focus (Altamirano et al., 2010). Conversely, people high in reflective rumination tend to exhibit less flexibility and have trouble shifting attention to a new task (Davis and Nolen-Hoeksema, 2000; Whitmer and Banich, 2007).

Whereas low rumination might be beneficial for athletes in football and field hockey, the same might not be true for participants in other sports. For example, it might be that higher levels of rumination are associated with success for people participating in more individual sports such as running. If participants in dynamic, team sport are low in rumination because of the need to be able to switch focus quickly, then the same relationship might not hold for participants in other, less dynamic sport. Similarly, previous research has found that personality differences are dependent on the type of sport in question (Plessner et al., 2009; Allen et al., 2011). Further research is needed to examine the impact of reflective rumination on other sports.

Along with examining only two sports, another limitation is that the results are correlational, not causal. Although it is possible that being low in reflective rumination leads to better performance in team sport, it could also be that competing in team sport somehow leads to lower levels of reflective rumination. It is also possible that some third variable, like the ability to switch quickly from task to task (Altamirano et al., 2010) or the ability to avoid the negative impact of anxiety producing situations (Mullen et al., 2007) is linked to both variables. Therefore, one avenue for future studies is to examine the relationship between expertise, reflective rumination, and other variables such as cognitive capacity and anxiety.

The relationship between rumination and athletic performance found here might also help explain why other personality variable have been linked with athletics. For example, previous research has found that athletic success is linked to higher levels of extraversion and lower levels of neuroticism (Allen et al., 2011, 2013; Allen and Laborde, 2014). As higher levels of extraversion and lower levels of neuroticism have been associated with lower levels of rumination (e.g., Luminet, 2003; Wupperman and Neumann, 2006), rumination might help explain this connection.

Though more research is needed to examine the relationship between rumination and performance in dynamic, team sport, the results indicate that this relationship exists and is strong. Athletes in these sports differed from non-athletes in their propensity to ruminate. The ever-changing way that competition can unfold might require that players not get stuck in repetitive thought patterns.

## Author Contributions

MR conceived and designed studies, analyzed data and drafted the manuscript. DM conceived and designed studies and contributed to the manuscript. AF designed and collected data for the field hockey players and contributed to the manuscript. JR designed and collected data for control group in field hockey study, contributed to the manuscript. JP designed study for field hockey players and contributed to the manuscript. BN analyzed parts of the data and contributed to the manuscript.

## Conflict of Interest Statement

The authors declare that the research was conducted in the absence of any commercial or financial relationships that could be construed as a potential conflict of interest.
