# Supplementary material for: Rumination and Performance in Dynamic, Team Sport
Source: Front Psychol. 2016 Jan 8;6:2016. doi: 10.3389/fpsyg.2015.02016 (PMC4705301; doi:10.3389/fpsyg.2015.02016)
Supplement: Supplementary file 1 [file Data_Sheet_1.DOCX]

Appendix

**Case Scenario 1: Respond with your reaction as if you were on the defense in the following scenario. Then proceed onto the next page for a short questionnaire.**

A foul was committed in the circle and the referee blows her whistle to signal a corner for the offensive team. The defense grabs their facial masks while the offense positions themselves on the circle and one player at insert. The referee signals that the corner can now take place. The insert positions herself and inserts the ball to the top of the circle. The defense rushes out to position themself. At the top of the circle one player traps the ball while another steps up and does a drag shot. The goalie slides onto her side with her stick extended covering the goal from side to side. An offensive player position to tap the ball around the goalie and a bang off the backboard indicates a goal and the offensive is up 1-0.

**Case Scenario 2: Respond with your reaction as if you were on the offense in the following scenario. Then proceed onto the next page for a short questionnaire.**

Now on the offensive, running down the field sprinting past the fifty-yard line. A few more yards and now pass two more players and now it’s two on one and the goalie. Just inside the circle and the defense not stepping up, one dodge around a defensive player, now just one on the goalie. Set up for a reversed chip shot, a shot notorious for its accuracy and power if done correctly. The shot is taken and the goalie goes down and extends herself to block the powerful shot. The player looks up and the ball is deflected off of the goalie’s padding and it rolls beyond the end line. The referee signals the ball out of bounds and the defense sets up to continue play. The other team is up 1-0.
